# Translating the International Code of Marketing of Breast‐milk Substitutes into national measures in nine countries

**DOI:** 10.1111/mcn.12730

**Published:** 2019-02-22

**Authors:** Isabelle Michaud‐Létourneau, Marion Gayard, David Louis Pelletier

**Affiliations:** ^1^ Department of Social and Preventive Medicine, School of Public Health Université de Montréal Montreal Quebec Canada; ^2^ Department of Family Medicine and Emergency Medicine Université de Sherbrooke Longueuil Quebec Canada; ^3^ Division of Nutritional Sciences Cornell University Ithaca New York USA

**Keywords:** advocacy, Africa, breastfeeding protection, International Code of Marketing of Breast‐milk Substitutes, policy process, Southeast Asia

## Abstract

The International Code of Marketing of Breast‐milk Substitutes (the Code) adopted by the World Health Assembly (WHA) in 1981 and regularly updated through subsequent WHA resolutions, represents the international policy framework for protecting breastfeeding against inappropriate marketing practices. By March 2016, at least 135 countries had some measures covering provisions of the Code in their legislation. The translation of the International Code into national measures was investigated in the context of the advocacy efforts undertaken by the Alive & Thrive (A&T) initiative with UNICEF and partners. A real‐time evaluation was carried out over 22 months in seven Southeast Asian countries (Cambodia, Indonesia, Lao People's Democratic Republic [Lao PDR], Myanmar, Thailand, Vietnam, and Timor‐Leste) and two African countries (Burkina Faso and Ethiopia). Drivers of policy change and progress were examined. Two theory‐based approaches were used: developmental evaluation and contribution analysis. Data collection methods included participant observation, key informant meetings, in‐depth interviews, reflective practice, and desk review. Overall, countries made significant progress in translating the International Code into national measures and in moving forward throughout the policy cycle. The main driver of policy change was the creation of a strategic group, which engaged key relevant actors and supported the government in the performance of 15 critical tasks, which the analysis reveals is a second driver. Those critical tasks are described in this paper and could help public health advocates to anticipate the stages and challenges of policy change and develop more effective strategies to translate the Code into their legal framework.

**Key messages**

- Countries experience challenges in translating the International Code of Marketing of Breast‐milk Substitutes into their legislation and in enforcing their legal measures.
- An advocacy approach involving a 4‐part process can help set and maintain the agenda for the Code throughout the entire policy cycle.
- Policy advocates have been able to support governments to carry out a set of 15 critical tasks by creating a strategic group and engaging key relevant actors.
- Constant vigilance against industry tactics is required throughout the policy cycle.

## 1 INTRODUCTION

Breastfeeding is one of the strongest evidence‐based practices for improving the health, development, and survival of children, with additional benefits for mothers, families, and society (Victora et al., 2016). In economic terms, the losses of not breastfeeding are estimated at about $302 billion annually (Rollins et al., 2016). Despite such solid evidence, globally, breastfeeding practices remain suboptimal; only 43% of children 0–5 months are exclusively breastfed (UNICEF, 2016). This reinforces the need to protect, promote, and support breastfeeding as stipulated in the Innocenti Declaration (World Health Organization [WHO] & UNICEF, 1990).

### 1.1 The International Code of Marketing of Breast‐milk Substitutes

The International Code of Marketing of Breast‐milk Substitutes (hereinafter referred to as “the Code”) represents the international policy framework to protect breastfeeding against inappropriate and unethical marketing practices from manufacturers and distributors of products under the scope of the Code. 1The Code applies to the marketing and practices related thereto, of the following products: breast milk substitutes including infant formula; other milk products, foods, and beverages, including bottle‐fed complementary foods when marketed or otherwise represented to be suitable, with or without modification, for use as a partial or total replacement of breast‐milk, feeding bottles and teats. It also applies to their quality and availability and to information concerning their use (Article 2. Scope of the Code, WHO, 1981). The Code was adopted in 1981 by the World Health Assembly (WHA) (WHO, 1981). It is regularly updated through WHA resolutions, and those should be considered together as far as the implementation of the Code is concerned (WHO, 2017a). In 2016, although at least 135 countries had some measures covering provisions of the Code in their legislation (WHO & UNICEF, 2016), its application remains challenging. Industry regularly circumvents the national measures and uses marketing practices that go against the Code (International Code Documentation Centre, 2017).

In 2014, the global sales value for infant and follow‐up formula was about $U.S. 44.8 billion, of which it is estimated that some 10% is likely spent on marketing and promotion (Smith, 2015). This sales value is projected to reach $U.S. 70.6 billion by 2019 (Rollins et al., 2016). Between 2008 and 2013, the East Asia and Pacific region had the highest volume increase, which was mainly driven by China, Indonesia, Thailand, and Vietnam (Baker et al., 2016). Given the profitability of this industry, the use of aggressive marketing practices is likely to increase, requiring governments to enact measures to protect their population against the deleterious effects of marketing on breastfeeding practices (Piwoz & Huffman, 2015; Sobel et al., 2011).

Research on the process of developing and implementing health policy in low and middle income countries has been quite limited (Gilson & Raphaely, 2008). This is also the case for the Code. There has been no empirical research on its full policy cycle, most studies focusing more narrowly on the challenges for its monitoring and enforcement (Aguayo, Ross, Kanon, & Ouedraogo, 2003; Parrilla‐Rodriguez & Gorrin‐Peralta, 2008; Taylor, 1998). Enforcement is known to be problematic, as seen in the widespread violations documented by International Baby Food Action Network (International Code Documentation Centre, 2017). However, to facilitate the translation of the International Code into national measures, a deeper and broader understanding of the other stages of the policy cycle is needed. The present study aims to fill this gap by presenting the results of a real‐time evaluation of the related collaborative advocacy efforts carried out by Alive and Thrive (A&T) in nine countries with UNICEF, development partners and local governments. The overall objective was to document the extent to which policy objectives were (or were not) achieved in each country and to identify the key drivers of policy changes.

### 1.2 Programmatic context

A&T is a 9‐year initiative to improve infant and young child feeding (IYCF) practices. After a first phase of implementation in Bangladesh, Ethiopia, and Vietnam, during which considerable gains in IYCF practices were achieved (Hajeebhoy et al., 2013; Menon, Rawat, & Ruel, 2013; Rawat et al., 2013), the Initiative received additional funding for a second phase (2014–2017). This second phase focused on sharing the successful experience of policy advocates in Vietnam to support governments and development partners in six countries within the region. The aim was to advocate either for the adoption of IYCF‐friendly policies or for the implementation, enforcement, or monitoring of existing policies. Countries included Cambodia, Indonesia, Lao People's Democratic Republic (Lao PDR), Myanmar, Thailand, and Timor‐Leste. At a regional workshop for Southeast Asia in 2014, each of seven country teams set specific policy objectives regarding three main areas: the Code, maternity protection, and health system strengthening. This phase also included two African countries, Burkina Faso and Ethiopia, which carried out advocacy efforts though their primary focus was on program implementation, and their work on the Code was less salient. In this paper, we focus on the Code and present the following: (a) key drivers of policy changes, and (b) major accomplishments achieved in the nine countries during Phase 2.

## 2 METHODOLOGY

### 2.1 Evaluation approaches

This real‐time evaluation used two complementary theory‐based evaluation approaches: developmental evaluation (DE) and contribution analysis (CA). DE supports the development and implementation of an innovation by collecting various types of data that help articulate feedback to adapt the innovation to the emergent and dynamic context (Patton, 2010). DE helped track the various actions in the nine countries and guide reflection on the advocacy efforts as they were evolving. CA allows for the assessment of whether an intervention contributed to the observed effects (Mayne, 2001, 2011, 2012; presented in a companion paper, Michaud‐Létourneau, Gayard, & Pelletier, 2019). CA acknowledges that factors other than the ones directly related to a project are at play and that they may significantly influence the outcomes. This was acknowledged for the evaluation of this Initiative. In sum, although DE helped primarily to collect various data and guide the reflection, CA helped to assess the contribution of various actions to the outcomes observed, utilizing the data collected using the DE approach. The ethics committees from the University of Sherbrooke and from FHI 360, the nonprofit human development organization that implement the A&T initiative, approved the research protocol.

### 2.2 Data collection

Table 1 details the data collection methods used between May 2015 and March 2017. No standardized data collection approach could apply to all countries, as the actions taking place and the actors involved varied. One researcher (IML) undertook four trips, visiting all but one country (Burkina Faso) to gather contextual knowledge and to identify potential informants for in‐depth interviews. Travelling with the A&T focal point of the different countries allowed for participant observation and made possible the documentation of strategies, tactics, and dynamics that would not have been captured otherwise. More specifically, the A&T focal point worked with partners and contractors on the execution of activities described in the country work plans. The observation by the researcher of those activities helped gain contextual knowledge and document the various ideas and actions undertaken by different actors. The researcher filled out a template identifying various items for each country: types of activities, objectives, participants, inputs, tactics or strategies, target audience, progress, comments, or questions. At the end of each trip, the researcher and the focal point took a time for debriefing and discussing some insights. Detailed notes were taken throughout the duration of the trip.

**Table 1:** Data collection

Methods	Description
Participant observation	Total: 7 A&T staff and representatives working with partners and local governments in 8 countries (all except Burkina Faso)
Key informant meetings in country	Total: 129 actors, 19 tape‐recorded interviews Cambodia (*n* = 11); Indonesia (*n* = 19); Lao PDR (*n* = 13); Myanmar (*n* = 17); Thailand (*n* = 9); Timor‐Leste (*n* = 13); Vietnam (*n* = 16); Ethiopia (*n* = 22); headquarter or regional (*n* = 9)
In‐depth interviews (calls and in‐person)	Total: 40 actors, 59 tape‐recorded interviews Cambodia (*n* = 3); Indonesia (*n* = 5); Lao PDR (*n* = 4); Myanmar (*n* = 5); Thailand (*n* = 3); Timor‐Leste (*n* = 5); Vietnam (*n* = 3); Burkina Faso (*n* = 3); Ethiopia (*n* = 9) Prospective: (a) document many aspects throughout the life of the Initiative, including activities, challenges and strategies to address them, contextual factors, and accomplishments, (b) track changes in real time (activities, contextual factors) and link them to outcomes. Retrospective: Further investigate potential triggers (key activities and contextual factors) that were flagged and found to be critical during the course of the evaluation process and also perceived outcomes.
Reflective practice	Living documents were developed to stimulate reflection with core actors on strategies to obtain validation (theories of change, exploration of concepts). They helped provide feedback and insights with different actors. They also helped in identifying less tangible outcomes (relationships, ideas, conditions for success) that could have contributed to trigger policy changes.
Desk review	A large number and diversity of documents were collected and reviewed to track activities, outcomes, contextual factors, and linkages. Research: opinion leader assessments, legal reviews, media audit reports; strategic documents: policy briefs, Code monitoring reports, one pager, joint letters; A&T resources: workplace lactation toolkit, advocacy guide; A&T working documents: donor reports, presentations, advocacy strategy, trip reports, internal briefs, concept notes, timeline; country teams instruments: roadmaps/workplans, meeting minutes; reports: regional workshops organized by A&T and UNICEF in 2013, 2014, and 2016; internet communication: email exchanges on various topics; exercises done by country actors: timetable by policy asks; documents on work definition: terms of reference (technical inputs from A&T on those types of documents); progress update: updates on the workplan, briefing notes, newsletters; field trips: agenda, notes; media outlet: opinion editorials, newspaper articles, TV spots; national policies, strategies and programs: IYCF, multisectoral work, nutrition; legal documents: official and draft Code from the various countries, labor code; international references for the Code: breaking the rules, Euromonitor, Code status report, NetCode; international references for maternity protection: ILO convention, ILO recommendation, ILO toolkit.
Participants included representatives from: coalitions (Thai Alliance for breastfeeding action, SUN—civil society coalition); donors (Irish aid, Millennium Challenge Corporation); foundations (Thai Breastfeeding Center Foundation, Alola Foundation); government (President's office, various departments of Ministry of health); headquarters and regional offices (A&T, UNICEF, Save the Children, WHO, IBFAN‐ICDC, FHI360); independent (A&T consultant); National Assembly Standing Committee; NGOs (Helen Keller International, Save the Children, World Vision, Helvetas—Swiss Intercooperation, Care, Plan International); research institutions (University Research Co., SMERU Research Institute, National Institute of Public Health, Timor research, National Institute of Nutrition, National Nutrition Center); United Nations agencies (UNICEF, WHO, FAO); university (Universitas Padjadjaran).	

*Note*. A&T, Alive & Thrive; FAO, Food and Agriculture Organization; FHI 360, name of a nonprofit human development organization; IBFAN, International Baby Food Action Network; ICDC, International Code Documentation Centre; ILO, International Labor Organization; IYCF, Infant and Young Child Feeding; n, number of actors; NGO, non‐government organization; SUN, Scaling up Nutrition; UNICEF, United Nations Children's Fund; WHO, World Health Organization.

Participants were identified either as primary or secondary. Primary participants were those most closely engaged with the advocacy efforts of A&T, UNICEF, and partners. For most of them, data collection involved several in‐depth interviews (up to a maximum of 7), which generally lasted between 60 and 90 min. Typically, the secondary participants were met during country visits; they provided knowledge about the overall context in the country and were involved with advocacy efforts to varying degrees.

### 2.3 Data analysis

All tape‐recorded interviews were transcribed verbatim. Thematic content analysis (Miles & Huberman, 1994) was carried out through an iterative process in which different types of coding (Saldaña, 2012) were performed with the QSR International's NVivo 11 software. Initially, two researchers (MG and IML) coded individually with cross‐checking (double coding) and ongoing discussion on the codebook. Once most categories had been developed, coding proceeded individually (MG) with discussion with the research team whenever needed. The notes and summary of main insights produced during each trip, based on participant observation, were other important data sources. Those primarily helped to document the various strategies planned in the different countries and allowed the researcher to follow‐up on the progress of those strategies. Meeting notes and informal discussion notes were also coded. Other types of documents were read, and the main elements were extracted to develop different pieces of analysis. Throughout, emerging themes were corroborated by triangulation from different data sources.

A systematic approach was carried out with most of the data collected. First, numerous elements (strategies, events, challenges, contextual factors, and accomplishments) were tracked throughout the evaluation, drawing upon all the available data at a given time. A chronology of events was thus developed for each country, which was completed with a desk review. The data collected from the nine countries were then organized according to the policy cycle (Clark, 2002). By taking all countries together, the full policy cycle was represented, and the main active stage, which was the latest stage on which actors focused their efforts during the period of this study, was clearly identified for each country. Finally, through this process, activities were identified that could be linked to certain outcomes and that helped to advance the policy cycle. Those activities were deemed “critical” activities, and the rationale for their identification was based on four criteria presented in Box 1. Throughout the evaluation, preliminary findings were validated with country actors, A&T focal points, or representatives.

**Box 1: Criteria for the identification of critical activities**

The activities meet at least one of the following criteria:

1. The activity has occurred in several countries and has been linked to an advancement within one or more stages of the policy cycle (common positive contributor).

2. The activity has been recognized to help in overcoming a challenge that hindered progress in the stages of the policy cycle (overcoming factor).

3. The activity has occurred in only one country but has clearly been identified as a trigger by several actors (strong contributor).

An additional criterion was used as a counterfactual to reinforce any of the above criteria:

4. An absence of this activity has been observed along with an absence of advancement in the stages of the policy cycle (counterfactual factor).

## 3 RESULTS

Findings from this real‐time evaluation based on the country experiences in nine countries are presented here organized in two parts: (a) two main drivers to translate the International Code into national measures, organized according to the stages of the policy cycle and (b) progress achieved on the Code. A terminology for key terms used in this section is presented in an Online Supplementary Material (OSM; Annex 1). In addition to these primary results, a composite case is provided in Annex 2 to illustrate in a more holistic way the experience of actors working to advance the Code, the challenges they face, the strategies they try to put in place, as well as some intermediate and major accomplishments. It also illustrates the non‐linear nature of the processes.

### 3.1 Main drivers and triggers of policy change according to the policy cycle

**Figure 1:**
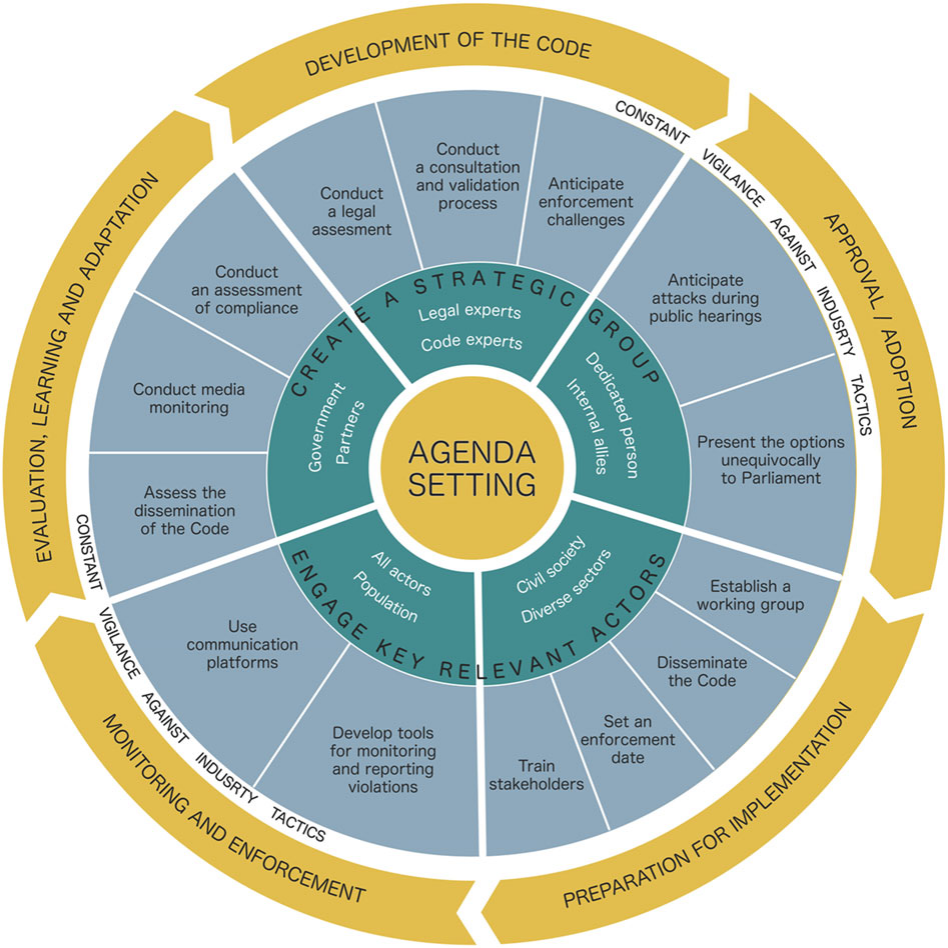
Critical tasks to translate the International Code into national legal measures

The different stages of the policy cycle observed included agenda setting, development of the Code, adoption, preparation for implementation, monitoring and enforcement, and finally, evaluation, learning, and adaptation. Although the stages of the policy cycle do not typically take place in a strict linear manner, this real‐time evaluation revealed that there was a certain sequence in terms of how progress from one stage to another was achieved. At the onset, the creation of a strategic group represented the first driver of policy change. At each subsequent stage, several activities were identified as critical to trigger advancement within the policy cycle. Later, those activities were termed as critical tasks: none of them was sufficient by itself to move forward the whole policy cycle. Nonetheless, the more those tasks can be carried out, the more likely a country is to progress within the policy cycle. Therefore, this set of critical tasks represents the second key driver of policy change. Those two drivers are represented in Figure 1 with an emphasis on each critical task according to the stage of the policy cycle.

#### 3.1.1 Agenda setting

The main driver of progress was the creation of a strategic group and the subsequent engagement of key relevant actors.

The creation of the strategic group was a direct result of the inputs from the advocacy efforts engaged by A&T, UNICEF, and partners (Michaud‐Létourneau et al., 2019). Using the A&T advocacy approach, the actors of the strategic group helped set and maintain the Code on various agendas at all stages of the policy cycle. This was demonstrably critical in ensuring the constant progress in the work. By engaging key relevant actors at appropriate stages, the strategic group also supported the government's efforts to realize the 15 critical tasks, thereby triggering progress throughout the policy cycle.

The various actors engaged in this process and their main contributions are described in Table 2. The various groups of actors engaged in the work had fluid boundaries and actors moved in and out, although the groups' functions or roles remained somewhat constant.

**Table 2:** Actors engaged in translating the Code into national measures

Groups of actors	A domestic group was composed primarily of local actors who represented different government entities and who were often directly involved with the law‐making process. It played a crucial role in the legal processes. A group of development partners was present in the country. A strategic group of actors was created as the convergence of these two previous groups. Its main function was to create and carry out strategies to advance the Code. It acted as a resource for the government: to connect with international experts, to have good relations with international organizations, to engage the right people at the right time.
	Existing technical groups such as the ones related to the Scaling‐Up Nutrition movement sometimes provided a platform for work around the Code, but this was not always the case.
	The collection of actors united their efforts throughout the processes to act together and counter various aggressive techniques by the industry.
Individual actors	Champions were instrumental in building consensus with other actors and carrying out certain activities. For example, strong advocates for the Code within the government were not afraid to stand up and defend the Code from tactics used by industry.
	Dedicated persons specifically in charge of follow‐up on actions to advance the Code in a country made it possible to remain alert and act quickly.
	Internal high‐level allies informed members of the strategic group on the internal processes of Code approval and alerted them when there was interference by the industry.
	Domestic legal experts who were well versed in the legal system facilitated the advancement of various stages, especially early on. They helped the various actors navigate through legal processes to work on the Code effectively or defend it whenever necessary. When legal actors did not have a background in health and/or on the Code, sharing evidence on the importance of breastfeeding and supporting them on problems related to the Code and its application appeared key.
	International Code experts (from UNICEF and International Code Documentation Center) frequently acted as external resources to support local actors. They were regularly mentioned as having played an important role in the development of Code drafts. In several countries, a consensus building or policy dialogue was organized and such experts were invited to: guide the staff of certain ministries on legal aspects related to the Code, share experiences from other countries, review the regulations related to a draft Code, and guide the local actors in finalizing the Code. The use of international legal experts has often proved decisive during the approval stage to present the facts and respond to criticisms from the industry.
	Actors from diverse sectors, for example, the Ministry of Culture or Ministry of Information were involved in work with the media, which proved to be effective.
	Researchers played a key role in generating evidence, providing supportive arguments and sometimes became champions.
	Civil society organizations helped raising awareness and monitoring violations; they also undertook advocacy activities.
	The population of some countries was engaged to encourage involvement with monitoring violations; however, such an activity was at an early state.

The ongoing advocacy efforts of the strategic group helped to place the Code on the agenda and to keep it there to achieve progress at any subsequent stage through the realization of the 15 critical tasks described below.

#### 3.1.2 Development of the Code

The Code can remain in development and/or stalled for several years. The regional workshops organized by A&T and UNICEF provided a unique opportunity for the actors of the various groups to refine and accelerate their efforts upon return to their country. They acquired more knowledge on the Code, interacted with international Code experts, learned from other countries, and strengthened their national team. The strategic actors of the different countries each used their own approach to develop the Code. Three activities were especially helpful.

1. Conduct a legal assessment: In countries where a legal review was carried out, strategic actors could tailor their approach based on crucial information: the hierarchy of the laws of a country, the frequency with which laws were revised, and the plan for future revisions. Such information allowed groups to examine whether provisions of the Code could be inserted into current laws, a less burdensome process than creating new ones. For example, in Vietnam, specific provisions of the Code were included in the Advertisement Law. Using existing laws not only simplified the process by providing a legal basis for developing subsequent sublaws, it also prevented the need to create a new enforcement mechanism from the start when one was already in place.
2. Conduct a consultation and validation process: An inclusive process for consultation and/or validation gradually helped to build consensus among the various actors while improving the draft Code. In some countries, actors who were not consulted beforehand, including medical associations or other health professions, were reluctant when the Code was finally presented to them and even opposed it.
3. Anticipate enforcement challenges: Formula companies regularly interfered with the work of the strategic group trying to dilute the provisions of the Code and weaken legislation. At this stage, actors had a chance to stay ahead of the industry, anticipating potential challenges rising at later stages by developing fines and penalties and their application.

For some countries, the development of the Code sought to replace an existing one that had been developed and approved in the past but was found to have several weaknesses. In such a case, the strategic group worked on the revision of the Code or on various pieces of legislation, but the process appeared similar.

#### 3.1.3 Approval/adoption

Seeking approval of the Code often was long and arduous depending on the internal processes of the country and nature of the opposition. The draft typically must pass through a series of revisions and approvals before final adoption. When a consultation process was carried out during the development phase, the various rounds of comments and negotiations on drafts appeared to facilitate the approval stage. Other activities were believed to further optimize this stage.

- Anticipate attacks during public hearings: At this stage, the comments of the opposition, generally formula companies, abounded. Some countries that had tried to introduce specific points in the draft regarding the application and monitoring of the Code had to remove them, which had a heavy impact on the later stages. It is therefore of prime importance that these sections of the draft be carefully thought out beforehand and solidly built, so that they could be firmly defended. One strategy was to have responses ready for the opposition's false arguments or complaints about the age range during which marketing should be restricted.
- Present the options unequivocally to Parliament: The way key actors presented the options to legislators influenced how they supported (or did not support) an issue. In Vietnam, a champion framed the voting options for the members of the assembly with a child right and people‐centred lens: “If you vote for Option 1, for a ban on advertisement for children under 24 months, you are voting for the benefit of the children, future generations, and human resources of this country. If you vote for option 2, for a ban on advertisement for children under 12 months, the beneficiaries will be the companies.” This unambiguous and binary way of presenting the options in favour of the Code was mentioned as a trigger for the team in Vietnam to win with an unprecedented majority.

#### 3.1.4 Preparation for implementation

At this stage, scenarios of violations of the Code are identified, and the desired responses and sanctions are defined. Countries can stay at this stage for a long time, but several activities helped to achieve progress.

- Establish a working group: The countries that established a working group to coordinate and oversee the work on the implementation of the Code made substantial progress. This group could deal with any issue raised, for example, by the formula companies who questioned the Code application at numerous points in order to delay enforcement. This task involved clarifying roles and responsibilities of various government entities, but in some countries, discussions also took place to identify potential roles for civil society to support the working group.
- Disseminate the Code: Various approaches were used, such as through meetings, the media, and/or letters sent directly to the formula companies. In Myanmar, as soon as a working group was set up for the implementation, a meeting was organized by the government, which made a clear and strong statement on what was expected of companies. In Vietnam, letters were sent to companies. Other actors within the government, as well as in the population, were sometimes informed about the Code for the potential role they could play.
- Set an enforcement date: Enforcement could occur when a fixed date for compliance was established. In Myanmar, rapid progress was made on several fronts. After approval of the Code, a working group was created who set the date for Code enforcement at 2 years after approval. Although this allowed for the actors to organize and for the structures to be adequately set up, it represented a long duration, which is not desirable.
- Train stakeholders: Regular orientations on the Code were undertaken for various types of actors, particularly health professionals. Past practices, such as the distribution of formula samples to mothers in hospitals, contravened the Code. Because a tactic regularly used by industry was to target various health professionals that reach mothers and families, it was emphasized that they needed to become familiar with the Code, the issues involved, and the role they should play to protect breastfeeding.

#### 3.1.5 Monitoring and enforcement (implementation)

This stage, which includes Code surveillance, is essential to achieve its ultimate objective: to regulate the actions of industry and ensure the compliance of health workers, media, retailers, and others for the well‐being of children. However, tremendous challenges were experienced. WHO, UNICEF, and other agencies had jointly developed NetCode to guide countries in creating their own monitoring system. 2WHO/UNICEF, NetCode Protocol—protocol for the assessment and monitoring of “the Code” and relevant national measures—summary, available at: http://www.who.int/nutrition/netcode/protocol_summary.pdf?ua=1
 Though few documents/tools related to NetCode were then available, agencies provided direct assistance in setting up this monitoring system, which appeared to enable progress.

- Develop tools for monitoring and reporting violations: Checklists were developed and used for ongoing monitoring. Cambodia had advanced on this subject and had developed four checklists for monitoring, based on NetCode resources: (a) points of sale, (b) audio‐visual promotion and advertising (television, radio, print material, online), (c) labels and packaging (for the marketing of products for IYCF), and (d) health facility promotional materials and activities. To report violations of the Code, some countries engaged civil society and the general population. In Myanmar, a phone application called Kobo Collect used by Save the Children allowed anyone to report violations, and this NGO used that data to create regular reports.
- Use communication platforms: Usually, it takes time before Code violators are penalized. To guarantee rapid follow‐up by the government, a reliable data management system was necessary to efficiently link the various bodies and ensure the transfer of information. Examples of how communication forums were useful to help the reporting system were also mentioned in some countries.

#### 3.1.6 Evaluation, learning, and adaptation

A second type of surveillance is evaluation. Per the NetCode definition, a periodic evaluation is required to verify the degree of adherence to national measures as well as to the International Code and to identify gaps and issues that need to be addressed through legislative measures (WHO & UNICEF, 2017). Of the countries included in this real‐time evaluation, few had reached this stage.

- Assess the dissemination of the Code: In some countries, studies were carried out to assess the dissemination of the Code. The sharing of their results helped to trigger actions to address gaps. For example, in Cambodia, the government, supported by an NGO, conducted an initial assessment in 2014 to investigate whether key stakeholders within the four key ministries were aware of the Code's provisions and to what extent these were implemented. With the results, stakeholders became aware of certain gaps and of the need to respond to them to reinforce Code compliance. This piece of evidence triggered subsequent actions. Periodic evaluation of the monitoring and enforcement system thus appears to be insightful and necessary.
- Conduct media monitoring: In most countries, the findings from media scans were consolidated into country reports and provided to policymakers (Vinje et al., 2017). In Myanmar, the results of the media scan were presented to the working group responsible for enforcing the Code; this group then requested a new audit to see if there were any improvements.
- Conduct an assessment of compliance: Some countries have assessed compliance of industry with the provisions of the Code and drafted reports. In Vietnam, in 2016, a large workshop involving many actors and several companies was organized in partnership with the Ministry of Health, during which results on compliance were disseminated and the provisions of the Code were recalled. This led to excellent discussions on how to strengthen the compliance of these companies with the provisions of the Code.

#### 3.1.7 Throughout the policy cycle

- Constant vigilance against industry tactics: The Code represents a threat to the industry whose interests and actions undermine breastfeeding. Companies will therefore use many tactics to hinder the development, adoption, implementation, or survival of the Code and will constantly try to weaken it. This appeared to be the case in Lao PDR in 2007, when the Code was downgraded due to the influence of industry. In Vietnam, mid‐2016, companies managed to open the vote concerning the ban on advertising for children under 24 months. They wanted to lower the age to 12 months, even though a May 2016 resolution of the WHA 69.9 referred to 36 months and urged governments to implement the new guidelines to end the inappropriate promotion of products for infants and young children. However, strategic actors were able to react quickly and to counter this offensive, preventing backsliding. These two examples illustrate the need to remain vigilant and convincing at all times. In the OSM, Annex 3 presents several tactics used by industry.

Remaining vigilant and keeping strategic actors informed of what was going on in the background appeared crucial. Some countries benefited from coalitions of actors who worked together to protect breastfeeding. The fact that they networked allowed them to exchange relevant information quickly, either through social media, email, or new technologies. Through such channels, they motivated themselves and organized concerted actions to counter the offensives of industry. When relationships were created with international experts or other resource persons, it helped actors to react quickly. Specific actions were sometimes undertaken within 24 hr, a required rate of response in certain circumstances.

### 3.2 Progress on the Code in nine countries

Table 3 presents the policy objectives set by each country team prior to this evaluation and some insights on progress. Several high‐level observations are noteworthy. First, prior to the regional meeting in March 2014, most countries had a Code in place. However, this version of their national Code had insufficient provisions or low ranking in the hierarchies of laws, which explains why many participants at the regional meeting said they wanted to strengthen it. Second, the policy objectives were broad and ambitious, for example, in proposing “revision, monitoring and enforcement” of a national Code. Third, none of the national Codes seemed to be enforced, allowing industry the freedom to continue advertising their products.

**Table 3:** Policy objectives and progress on the Code by country

Country	Past accomplishments	Policy objectives after the Bangkok meeting in March 2014*	Selected key accomplishments since the Bangkok meeting
Cambodia	In 2005, subdecree 133 was approved. In 2007, the joint Prakas 061 were approved.	Effective enforcement of existing legislation, subdecree 133 focusing on BMS, through oversight mechanism and penalties' regime Amendment of legislation to differentiate between labeling and promotion standards for BMS and complementary foods	*Preparation for implementation* Creation of oversight board, executive working group, and control committee (2014) Implementation guidelines and TOR approved by MOH (December 2015) Consensus on the monitoring forms (4 checklists; May 2016) *Monitoring and enforcement* Monitoring of the Code and testing/evaluation of the system in 4 provinces (2017) Spot check visits for national and subnational levels
Indonesia	The national Code (1997) was translated into different regulations. Parts of the Code were inserted into the food label and advertisement regulation supervised by BPOM. The food law was revised in 2013, which required revision of various laws and regulations related to the Code.	Extend labeling and advertising government regulation to cover BMS up to the age of 2 years	*Agenda setting* MOH led a strategic team to work on the national Code (April 2016) Declaration of the government to support WHA resolution 69.9 (August 2016) *Development of the Code* Food standardization unit (under BPOM) is revising the Code (December 2016) and the Office of the President is highly involved
Lao PDR	In 2004, a Code is in place. In 2007, the Code was downgraded to a voluntary agreement on IYCF products—control and provisions were weakened.	Strengthen regulation of private companies and BMS Code Promote awareness of existing BMS Code, including Code violation and importance of protecting breastfeeding, to relevant government bodies	*Agenda setting* Event with Inter‐Parliamentary Union: The Code was mentioned as a priority (November 2014) *Development of the Code* Intersectoral task force was formed (December 2016) Draft of Prime Minister's decree (August 2017) Smaller team is working on the draft Plan to submit it for approval in January 2018
		Revision, monitoring, and enforcement of national Code*	
Myanmar	No regulation existed regarding the Code.	Revision, monitoring, and enforcement of national Code*	*Agenda setting* Country team who attended the 2013 April workshop worked on the Code afterwards *Development of the Code* Support from UNICEF and ICDC to draft a Code *Approval/adoption* A national order of marketing of formulated food for infant and young child was approved in 2014 *Preparation for implementation* Creation of the technical working group for monitoring and enforcement (November 2015) Dissemination workshop for companies (December 2015) Training Public announcements Clarification of the roles of various institutions (March 2016) *Monitoring and enforcement* Official deadline compliance with the National Order was set (initial date of July 2016 reset later to November 2016) Routine reports on the Order's violations began to be submitted to the government (July 2016)
Thailand	In 2008, a first Code was adopted; however, it was neither a law nor regulation.	Improve exclusive breastfeeding BMS Code act*	*Approval/adoption* State council approved a new draft of the Code (December 2015) Passed the first hearing of the National Legislative Assembly (November 2016) Final approval (April 2017)
Timor Leste	In 2009, the draft Code was revised, but never presented for approval.	No specific policy objective regarding the Code set at the 2014 meeting	*Agenda setting* New revision planned for 2017
Vietnam	In 2012, the Advertisement Law was approved; it banned advertising BMS up to 24 months of age, complementary foods for children under 6 months of age as per the BMS Code, feeding bottles and teats.	Enforcement of provisions in the Advertisement Law regulating BMS marketing	*Approval/adoption* In 2014, decree 100 approved to further specify the Advertisement Law. *Preparation for implementation* Dissemination workshop for health staff (2014) Letter to industry Training workshops *Monitoring and enforcement* Media monitoring Fixed visits and visits upon violations Inclusion of decree 100 compliance into the National Hospital Quality Criteria and accreditation system (2016)
Burkina Faso	Agreement (limited information)	Support efforts to strengthen the national BMS Code	*Development of the Code* Support from IBFAN and UNICEF to develop a draft Revision of the draft decree (January 2016) Validation workshop supported by A&T (October 2016) *Approval/adoption* Several steps are required before approval Actors hope to have a Code approved by the end of 2017
Ethiopia	In June 2011, the food advertising directive was enacted.	Strengthen the government's efforts to create an enabling federal and subnational environment for improved IYCF services (no particular focus on the Code)	*Development of the Code* The EFMHCA has adapted the Code Support from UNICEF *Approval/adoption* Infant formula and follow‐up formula directive (March 2016) *Preparation for implementation* The nutrition donor forum wants to do a “bottleneck study” to examine why the Code is not implemented (2016)

*Note*. A&T, Alive & Thrive; BMS, Breastmilk substitutes; BPOM, *Badan Pengawas Obat dan Makanan*, the equivalent of a national Food and Drug Administration in Indonesia; EFMHCA, Ethiopian Food, Medicine and Health Care Administration and Control Authority; IBFAN, International Baby Food Action Network; ICDC, International Code Documentation Centre; IYCF, Infant and Young Child Feeding; MOH, Ministry of Health; TOR, Terms of reference; WHA, World Health Assembly.

For Southeast Asian countries, the policy objectives were agreed at the 2014 Bangkok meeting.

* Countries in Southeast Asia that have modified slightly their policy objectives after the 2014 Bangkok meeting.

For the African countries, policy objectives came from their Detailed Implementation Plan.

Countries made significant progress in translating the Code into national measures and advancing through the policy cycle. Three countries were working on the revision of their Code (Indonesia, Lao PDR, and Burkina Faso) after putting it on the agenda of various organizations (governmental and non‐governmental). Thailand had progressed through the internal approval process for its Code, which was finally approved early in 2017 after the end of this evaluation. Cambodia, Myanmar, and Vietnam progressed with Code monitoring, including creating guidelines, setting up working groups (oversight board, control committee, executive working group), or adopting a deadline for compliance. In Vietnam, a decree was approved regarding the marketing and use of feeding products (feeding bottles, teats, and pacifiers) for young children, to further specify the Advertisement Law. Timor Leste had faced numerous challenges but was gaining momentum in the critical first step of setting the agenda and engaging key relevant actors. The differential progress across countries is due in part to the long timeline typically required for policy change as well as to variation in the opportunities, facilitators, and barriers.

Examining major accomplishments to assess progress is essential, but it is equally important to document and recognize intermediate accomplishments to more fully assess progress. Annex 4 highlights a substantial number of intermediate and some major accomplishments regarding the Code. It is important to note that the distinction between those two types of accomplishments is not always easy to draw; typically, smaller wins are the ones that lead to bigger wins. Some strategies also appear in intermediate accomplishments, especially when we were able to link them to the bigger wins. This annex shows that intermediate accomplishments are more numerous than major ones. Therefore, relying solely on major accomplishments to assess progress does not do justice to all the work done and does not help in understanding what it takes to reach major accomplishments.

Finally, Vietnam became a model, showing it is indeed possible to make progress using various strategies. The experience of Vietnam, shared at the regional workshop, catalysed movement in all six countries in the region by motivating actors to embark on a similar journey, which is a major accomplishment in itself.

## 4 DISCUSSION

In this paper, we present a broad range of findings on the progress achieved in nine countries in which the A&T initiative took place to improve IYCF policies in collaboration with UNICEF and other partners in supporting governments. We highlight two major contributions and several implications.

The first contribution regards the set of 15 critical tasks, which were specific to the various stages of the policy cycle and that help trigger progress on the Code. Despite calls for coordinated global action to address the challenges surrounding the promotion of breastmilk substitutes (McFadden et al., 2016), a detailed understanding of how to achieve progress at the country level is still poor. In the WHO 2011 status report on country implementation of the Code, an area identified as needing further efforts was “clarity on processes necessary for the adaptation of the Code” (p. vii). It was also suggested that for the successful implementation of the Code, it was critical to have “political commitment and advocacy” and “a critical mass of advocates” (WHO, 2013). The latest report does not bring more clarity on such processes (WHO & UNICEF, 2016). In the present paper, we identify critical tasks within this process and locate them within the various stages of the policy cycle that advocates must manage when trying to translate the Code into their national legal framework. The findings can help them anticipate subsequent stages and challenges and develop more effective strategies to influence various elements of the policy process.

To our knowledge, this is the first study to examine dynamically and prospectively the full policy cycle regarding translation of the Code into national measures and to follow in real‐time key actors engaged in this work in multiple countries. Some of the existing literature presents the history of the Code (Bar‐Yam, 2001; Brady, 2012), which is valuable to ensure that new generations of health professionals understand why it was created and its continued relevance as well as their role in protecting breastfeeding. However, much of the literature draws attention to problems and focuses on one stage of the policy process (especially monitoring and enforcement) because of the many reports of industry violations of the Code (Aguayo et al., 2003; Costello & Sachdev, 1998; Parrilla‐Rodriguez & Gorrin‐Peralta, 2008; Pereira et al., 2016; Perera, 1999; Pries et al., 2016a, 2016b; Sweet et al., 2016; Taylor, 1998). Notably lacking is attention to the agenda setting and adoption stages. UNICEF did conduct a landscape analysis on political commitment (UNICEF, 2013), but its focus was broader than the Code, and its intent was not to understand how to increase political commitment to the Code. A number of other papers provide broad recommendations and lessons learned based on experiences (Barennes, Slesak, Goyet, Aaron, & Srour, 2016; Sokol, Clark, & Aguayo, 2008), but they do not go into practical details on the strategies and activities that can help translate the Code into national measures. Just recently, WHO and UNICEF shared a protocol for ongoing monitoring systems, a much needed NetCode guidance (WHO, 2017b). The findings reported here thus fill an important gap by providing guidance to breastfeeding advocates regarding the multiple stages of the Code policy cycle and the critical tasks at each stage that can help them advance within the policy cycle.

The second contribution regards the assessment of progress. The creation of a strategic group, thanks to advocacy, and the subsequent engagement of key actors relevant to each stage of the policy cycle was found to be a major driver of progress. Although it is widely recognized that the stages within the policy cycle do not occur in a linear fashion, agenda setting often is conceived as being the first step, which enables progress in the subsequent stages. However, this study demonstrated that agenda setting efforts are needed throughout the policy cycle. It also demonstrated the need for ongoing advocacy efforts—such as the ones supported by A&T, UNICEF, and their partners—and the need to constantly revise strategies in light of the changing context and opportunities. In turn, this required policy advocates to have some flexibility in order to be able to recognize and seize opportunities and to react to emergent conditions. Moreover, the presence of key actors with “strategic capacity” (Pelletier, Menon, Ngo, Frongillo, & Frongillo, 2011) helped to achieve progress. Actors from the strategic groups could tailor their advocacy messages based on diverse forms of evidence.

These findings have immediate and practical implications for the Global Breastfeeding Advocacy Initiative led by UNICEF and WHO (Taqi, 2014; World Health Organization‐UNICEF, 2015) now called the Global Breastfeeding Collective (https://www.unicef.org/nutrition/index_98470.html) and that seeks to create a social movement to make breastfeeding the social norm, including implementation of the Code (Arts, Taqi, & Bégin, 2017). Although it is recognized that advocacy and policy change efforts can be informed by diverse theories from political science, sociology, and communications (Stachowiak, 2013), the findings presented here add to a recent focus on generating and using “practice‐based evidence” (Green, 2008; D. Pelletier et al., 2013).

The findings have implications on how progress can be achieved in other regions. As a result of the advocacy efforts, most of the countries in Southeast Asia in this study have now prioritized work around the Code. This has triggered important momentum in the Southeast Asian region as a whole. It also explains why the great majority of the countries followed in this real‐time evaluation have made significant progress in their policy process regarding the Code. One important driver that appeared to be an achievement by itself was the creation of a strategic group. Actors from the governments of those countries have really taken on the responsibility of improving the Code in their country and collaborate with development partners to achieve their common goal. African countries could benefit from the experience gained in Southeast Asia regarding the Code.

Finally, it is important to remember that the Code was intended to be adopted by all governments as a minimum requirement. Although many countries have made progress, their national measures do not always fulfil this requirement. Thus, additional work is needed to ensure that all the provisions of the Code and of the subsequent resolutions of the WHA, become part of their legislative framework in order to prevent inappropriate marketing of breastmilk substitutes and to protect child health. Of importance is the emergent challenge of advertisement through social media, which will require new strategies to monitor and enforce the Code (Abrahams, 2012), a stage, which continues to be a challenge in most countries. The guidance provided through the NetCode has been useful to country actors and warrants continued development. The case studies currently taking place in the scope of the NetCode should also provide much needed guidance in this area and complement the findings presented from this real‐time evaluation.

## ETHICS APPROVAL

Ethics committees at both the University of Sherbrooke and FHI360 approved the research protocol.

## CONFLICTS OF INTEREST

All authors confirmed that they have no conflict of interest related to the content of this paper.

## CONTRIBUTIONS

IML designed the real‐time evaluation, played a leadership role throughout all stages of the study, collected most data, and conceptualized the paper. IML and MG conducted data analysis, interpretation of results, and drafting of the different sections of the manuscript. DLP participated in data collection, advised at all stages of the process and collaborated in revising earlier drafts of the manuscript. All authors read, commented, and approved the final manuscript.

## FUNDING

Alive & Thrive is funded by the Bill & Melinda Gates Foundation, the governments of Canada and Ireland and is managed by FHI 360.

## Supporting information

Annex 1: Terminology
Annex 2: An illustrative case of the experience with the Code (annotated with terminology)
Annex 3: Tactics used by industry
Annex 4: Intermediate and major accomplishments regarding the Code per country
