# Supplementary material for: Translating the International Code of Marketing of Breast‐milk Substitutes into national measures in nine countries
Source: Matern Child Nutr. 2019 Feb 22;15(Suppl 2):e12730. doi: 10.1111/mcn.12730 (PMC6519018; doi:10.1111/mcn.12730)
Supplement: Supplementary file 1 — Annex 1: Terminology Annex 2: An illustrative case of the experience with the Code (annotated with terminology) Annex 3: Tactics used by industry Annex 4: Intermediate and major accomplishments regarding the Code per country [file MCN-15-e12730-s001.docx]

**Online Supplementary Materials**

**Annex 1: Terminology**

- ***Policy asks, policy objectives or expected results:*** In the context of the A&T and UNICEF initiative, those terms were used interchangeably and refer to the objectives that the country teams that participated in the 2014 Bangkok meeting have prioritized to focus on in the following years.
- ***Strategies*** are different types of actions that may be planned well in advance or be created following an opportunity in order to achieve specific outcomes. They become triggers when we are able to link them to progress.
- ***Challenges*** are different types of difficulties that are experienced related to the work, including barriers, bottlenecks, problems, or gaps (e.g. lack of people trained in advocacy).
- ***Drivers*** are factors, activities or strategies that are necessary to conduct to some outcomes or accomplishments.
- ***Intermediate accomplishments*** are outcomes that may appear intangible and of small importance (e.g. relationship created among partners), but that are often a pre-requisite for the attainment of a major accomplishment.
- ***Major accomplishments*** are often tangible outcomes that are easily recognized as they are the most expected ones for which actors strive for (e.g. national law, policy, strategy or plan).
- ***Triggers*** are factors, activities or strategies that can initiate or precipitate the achievement of some outcomes or accomplishments.

**Annex 2: An illustrative case of the experience with the Code (annotated with terminology)**

| Pacifica is typical of countries that have recently moved from a low to middle income country. Despite the economic growth of recent years, the prevalence of various types of undernutrition among children under five remains high. Since 2006, the country has created some regulations about the marketing of breastmilk substitutes as well as a voluntary agreement to protect infant and young child feeding ***(major accomplishments)***. However, breastfeeding rates continue to decrease, which leaves little doubt about the effectiveness of those measures. Formula companies have continued to gain ground through aggressive marketing. Unfortunately, successive turnovers within the government have delayed putting protection of infant and young child feeding on the political agenda ***(challenges)***. At the instigation of various health organizations that have had good relations with the government for many years, and following numerous advocacy workshops, the new Minister of Health has decided to commit himself to aligning with international standards ***(intermediate accomplishment)***. The Minister mandated the Chief of the Department of nutrition to accelerate the work on the Code. This led to the creation of a strategic group of actors working on the issue ***(driver)***.  After an initial missed opportunity in 2011, when the Food Act was revised, the recruitment of a legal expert ***(strategy)*** has helped better determine the best timing and optimum way in which a piece of legislation could fit into the legislative landscape: a sub-decree seemed the best option at that time. A first draft was therefore developed. Unfortunately, before its approval, the text received strong opposition from the companies but also from medical associations at the public hearings ***(challenge)***. The draft had to be reworked, and the assistance of an international expert on the Code, as well as the holding of a consultation process, was necessary in order for the different stakeholders to reach a consensus ***(strategies)***. The new sub-decree was finally promulgated in December 2012 ***(major accomplishment)*,** and immediately followed by dissemination workshops. Several trainings were also initiated in the first quarter of 2013 ***(intermediate accomplishments)***.  In the year following the approval of the sub-decree, although inspections were regularly carried out by the Ministry of Health, there was no other form of monitoring. As no deadline for compliance had been formally established, sub-optimal practices persisted in health facilities, and companies continued to violate the Code. Companies took advantage of the lack of precision regarding certain measures of the sub-decree, and corruption was present - affecting some government agencies. This prevented the establishment of a committee to be in charge of the application of the Code, which was planned at the onset ***(challenge)***. Fortunately, the strategic group represented a true sentinel of the Code, and prevented the sub-decree from being downgraded into an agreement. This group was able to carry out actions promptly and provided strong support to the Ministry of Health. For example, when companies exerted pressure on the government, through the Chamber of Commerce and by subtle pressure through direct access to the Prime Minister ***(challenges)***, the group organized letters that were sent to high-level actors within the government to support the Code and explain why the Code was important for the whole society ***(strategy)***.  In 2014, several study reports showed that violations persisted and several months of advocacy were still needed before monitoring and enforcement mechanisms could be developed. During this period, the process was slowed down by major droughts that caused famine in the south of the country and shifted the priorities of the government and the efforts of United Nations (UN) agencies and NGOs ***(challenges)***. In November 2014, almost two years after the approval of the sub-decree, a technical group responsible for the implementation of the sub-decree was finally set up ***(major accomplishment)***. Through regular meetings, several important steps were achieved: creation of a structure that included a committee linked to an enforcement mechanism and development of checklists for inspection ***(intermediate accomplishments)***.  Despite those efforts, there were still several gaps in the surveillance system: it was particularly difficult to trace violations and report them to the authorities in charge of enforcement ***(challenges)***. After the World Breastfeeding Week, in August 2016, and following the example of a neighboring country, the Pacifica government has allocated funds for a telephone line to report Code violations and for the development of a mobile application ***(intermediate accomplishment)***, allowing direct reports of violations, which had been difficult to do prior. In this way, violations can now be reported to the responsible authorities and monitoring is conducted on a large scale ***(major accomplishment)***. In December 2016, a new awareness-raising activity targeted health workers and the public through training sessions on monitoring, in an attempt to make the system more effective ***(strategy)***. The NGOs were of great support to the Ministry of Health at this stage.  Finally, a pilot study currently being carried out in three selected provinces will highlight the effectiveness of the system and make further improvements ***(strategy)***. The latest media monitoring carried out between September and December 2016 showed very encouraging results ***(intermediate accomplishments)***. However, there is a critical need to continue vigilance because the tactics of the formula companies are increasingly insidious. |
| --- |

**Annex 3: Tactics used by industry**

| **Tactics** | **Illustrative examples** |
| --- | --- |
| **Put pressure on government** | In many countries, the industry has direct access to, or has a significant influence on, for example, high-ranking government officials due to their important economic weight in the country. |
| **Use persuasive but incorrect arguments** | Companies can use false arguments to frighten the government and dissuade it from voting in favor of the Code. For example, a common false argument: "The Code is a violation of the free trade agreement. The case will be brought before the World Trade Organization." |
| **Gain a place in groups supporting the adoption of the Code** | In the various rounds of approval of the Code that precede adoption, companies attempt to take part in the consultative process in order to remove provisions from the Code and weaken it. |
| **Support medical associations or other health professionals’ associations** | The industry funds some medical or other health professionals’ associations, support them in organizing forums, provide information or take advantage of them to distort the true nature of the Code and minimize the risks of not breastfeeding. |
| **Delay the process** | This stratagem allows companies to sharpen their arguments. |
| **Use third parties to defend their interests** | Companies are sometimes supported by third parties to the government. Their communication channels often pass through chambers of commerce representing the economic interests of the various countries (eg EuroCham or AmCham). Other organizations defending companies may also be used (eg USABC). |
| **Promoting in a roundabout way** | Some companies do not promote the products directly targeted by the ban, but strongly encourage products, which have the same brand, the same logos, and the same appearance as the infant formula (cross promotion). Other times, they provide free samples to medical institutions. Companies also send their representatives to points of sale to talk about their products. |
| **Using social media and new technologies** | Companies take advantage of these forms of media and technologies, using them in a sophisticated and difficult to control manner. On the one hand, they do not publish images of the product that would constitute violations. On the other hand, they only provide part of the information and encourage consumers to order or ask for the rest of the information. They also send text messages to consumer phones. |
| **Sponsor government actors** | Some companies manage to circumvent the law by bribing members of the government. This allows them, for example, to contravene the law on labeling without suffering consequences. |

**Annex 4: Intermediate and major accomplishments regarding the Code per country**

| **Country** | **Strategies/intermediate accomplishments** | **Major accomplishments** |
| --- | --- | --- |
| **Cambodia** | 2014   - Situation analysis in Phnom Penh to assess to what extent the enforcement of Sub-decree and Joint Prakas was taking place and if key actors knew about them. After the results, the government met with formula companies. Letters were sent to health facilities to say that penalties would be given if they continued to distribute free samples.   Dec 2015   - Approval of the TOR for the oversight board, the executive working group and the control committee. - Approval of Guidelines for the implementation, monitoring and enforcement of Sub-Decree 133 and Joint Prakas 061.   April-May 2016   - The government and partners developed 4 checklists (one per line ministry) and a 1-week workshop was carried out to get consensus on the content.   Aug 2016   - Capacity building and orientations for Sub-Decree 133 were conducted by HKI to all the NGOs that work on IYCF at the provincial level.   Oct 2016   - The MOH conducted a session on the monitoring of Sub-Decree 133. - A 5-day workshop was conducted at MOH to discuss the draft of inspection and monitoring tools (national level). - 3-day meeting of the 4 line ministries with HKI and WHO in Sihanoukville (province) to discuss the training and content of the inspections. - Orientations at the national level (aligned ministries), as well as at the sub-national level (four provinces selected to conduct the pilot of monitoring and inspections system) conducted by MOH, and supported by HKI and WHO. - Training on Sub-Decree 133 for the media outlet organized by HKI and conducted by MOH. After that, actors mentioned that coverage on IYCF and the sub-decree has been increased.   Nov 2016   - Meeting between key stakeholders for the enforcement of Sub-Decree 133 (including MOH, WHO, UNICEF).   First quarter of 2017  -Spot check visits at the national and subnational levels to determine gaps in the monitoring process and when the violation reports are filed. | Aug 2014   - Creation of an oversight board that led to the creation of a proper mechanism to ensure enforcement. A focal point from each line ministry was assigned to it. - Creation of the control committee and the executive working group.   May 2016   - Consensus on the checklists for the monitoring of the Code - Preparation for the monitoring of the Code |
| **Indonesia** | March 2016   - The launch of the Lancet Series on Breastfeeding and the presentation of the “Cost of Not Breastfeeding” took place during the National Nutrition Day - identification of high-level champions and triggering event for more actions.   April 2016   - A large delegation attended the Regional Policy Workshop in Bangkok, strengthening the Code team. Afterwards, a group is started on WhatsApp for the participants to maintain communication. - MOH organized several follow-up meetings with the strategic group to move things forward for the Code.   May 2016   - MOH attended the meeting for WHA Resolution 69.9 and the Government of Indonesia has endorsed this resolution.   Sept 2016   - UNICEF and World Vision managed to join the public hearing organized in Makassar city in south Sulawesi, and inform the rest of the coalition on the developments.   Jan 2017   - Five health and nutrition organizations launched a joint statement regarding WHA Resolution 69.9. They recommended the government accept recommendations 1-5, and 7. However, they recommended to disagree with recommendation 6, arguing that the number of health providers requiring capacity building in IYCF is too large for the government budget, and that partnership with formula companies is possible.   Feb 2017  - Policy audit by HKI identified what specific policy actions need to be taken. | August 2016   - World Breastfeeding Week during which the Director General mentioned in front of a large forum that the Government of Indonesia supported the WHA Resolution 69.9.   December 2016   - The Food Standardization Unit (under the BPOM) was working on the revised draft of the Code. |
| **Lao PDR** | March 2014   - Joint UNICEF-WHO-SC-ICDC technical support mission to MOH in the area of Nutrition Law Development to assist Department of Health and Hygiene Promotion (DHHP) within MOH to define scope of nutrition law and assess the need to strengthen Code implementation in Lao PDR; share other country experiences; help formulate a road map for the way forward.   Nov 2014   - Inter-Parliamentary Union event held in Vientiane: one of the priority actions was “improved implementation, monitoring and enforcement of the International Code of Marketing of Breastmilk Substitutes and all relevant subsequent WHA resolutions, through the adoption of legally enforceable legislation.” - Law making circle in Lao PDR happens every five years (… 2010, 2015, 2020…). Instead of waiting until 2020 for a law, a government decree is considered because it is stronger than a regulation.   2015  - Regular meetings between the director of DHHP and UNICEF, SC and A&T to discuss the Code.  April 2016  - A&T conducted a legal review of regulations on breastfeeding protection, BMS and others including the law-making process and key government stakeholders.  June 2016   - A national consultant with legal background was identified and put in the MOH with the help of A&T. He helped them analyze the current situation of the Code and BMS policy making process in Lao PDR, but required support with specific Code-related issues.   August 2016   - Field trip to Vietnam with a delegation of 25 actors from MOH and from 4 provinces (however, for the SBCC work many actors are also involved with the Code).   July-Oct 2016   - DHHP has organized 2 meetings with key stakeholders (UNICEF, SC and other line ministries to talk about the Code). - A draft of TOR for a Task Force is developed.   Jan 2017   - First meeting of the Task Force took place in which a first draft of the revised Code is discussed. | Nov 2014   - High-level commitment to strengthen the Code   Jan 2017   - Draft of a Prime Minister’s decree for the Code |
| **Myanmar** | March-Aug 2015   - Media scan done by A&T.   Dec 2015   - The government disseminated the Order to the formula companies in a workshop, announcing that they needed to make changes to their practices to abide.   2016   - Training on new national community-based IYCF guidelines has been rolled out and involves an important champion, a renowned pediatrician. She integrated BMS code training and awareness within this training. - Three meetings of the TWG to discuss preparation for implementation.   Jan-March 2016   - The Surveillance Unit conducted a market survey.   March 2016   - SC and SUN-CSA monitor a database of BMS Code violations (through KoboCollect) and submit routine reports to government. - SUN-CSA is rolling out training on the BMS Code and on how to monitor to 5 community-based organizations in Magway, Chin, Rakhine and Shan.   May 2016   - Second official meeting of the TWG took place. A deadline for “voluntary recall” of products violating the BMS Code was set. - SUN stakeholders have jointly advocated and developed Code policy briefs on nutrition. It has reached the highest level of the new Government administration.   June-July 2016   - The FDA announced the deadline in a local newspaper, conducted market surveillance, and communicated with formula companies to assess their readiness. - Based on the response from formula companies, the deadline was extended until November to allow companies to sell or remove all products that were legally imported before the law went into effect. - SC and the SUN-CSA began to produce monitoring reports quarterly, in a new format for the TWG, and all members of the SUN networks. | 2014   - A National Order of Marketing of Formulated Food for Infant and Young Child was approved under the National Food Law.   Nov 2015   - A National Technical Working Group (TWG) was established as the official national government body charged with the design and oversight of the overall process for monitoring and enforcing the National Order. FDA, NNC, attorney general's office are part of this TWG.   2016   - Official deadline for “voluntary recall” of products violating the Order (Code): 24 July 2016. - Revision of the deadline to November 2016. - Monitoring reports begin to be produced and sent to the TWG. |
| **Thailand** | September 2014   - The Thai Alliance for Breastfeeding Action (TABFA) was created and the mission included mobilizing public policy advocacy to protect, promote and support breastfeeding and complementary feeding.   Aug 2015   - The revised draft circulated between different ministries for comments before going to the Cabinet.   2015-16   - International organizations sent letters to high-level government officials to advocate for the importance of supporting the Code to counteract pressure from formula companies on members of the government.   Jan 2016   - The Global Nutrition Report was launched in Bangkok, showing that Thailand was off course on all the WHA nutrition indicators. Surprised by those, the MOH committed to take action.   March – April 2017   - Media and breastfeeding orientation workshops organized by A&T, UNICEF and Thai Breastfeeding Center Foundation with broader aim of strengthening public communication on IYCF. A workshop with health professionals was held to discuss the BMS Code and encourage participants to share pro-breastfeeding messages with influential policy makers. A joint op-ed authored by A&T, UNICEF and Thai Breastfeeding Center Foundation was placed on the day of the vote to highlight the critical importance of passing the legislation. | Dec 2015   - The State Council has approved the draft of the BMS Code as a law (with the ban for up to 24 months). However, oppositions from medical associations have compromised and paralyzed the approval process (they are against regulating above 12 months).   2016   - Commitment of the MOH to improve the breastfeeding rates   2017  - BMS Code Act passed with more than 90% vote |
| **Timor Leste** | April 2016   - Regional Policy Workshop in Bangkok: the BMS code was raised as a priority among the country team members who attended.   June 2016  Considering that the draft was revised in 2009 and that it may require additional revisions, UNICEF was planning to hire a local attorney to review the law and compare it to make sure that the BMS code is up-to-date and follows all the current standards. Further delays took place. | Jan 2017  The BMS Code seems on the agenda of UNICEF Timor Leste for this year and other actors. |
| **Vietnam** | December 2014   - 3 national dissemination workshops took place to train and inform everyone on the contents of the Decree 100. - The legislation department sent the Decree 100 to all concerned agencies and to the formula companies.   April 2015   - Training of actors at the national level (all the health sector leaders, hospitals leaders, inspectors, health staff) by experts on Decree 100, violations, and monitoring. - Initiated the process of rolling out trainings in provinces. Joo Kean from the International Code Documentation Centre (author of the Code monitoring document – *Breaking the Rules*) was invited by A&T for the training of trainers.   Feb 2016   - The director of NIN (after it was reported that his staff violated Decree 100) organized a training workshop and invited A&T and the Legislation Department to come to train all their staff on Decree 100 and International Code.   May 2016   - The Legislation Department of MOH wanted to develop a monitoring mechanism, like a hotline, to receive reports on violations of Decree 100, but funding appeared a limiting factor.   July 2016   - Access to Nutrition Foundation (ATNF) developed a report on company's compliance to International Code and carried out a dissemination workshop. A&T linked ATNF and legislation department of MOH and they organized it with the support of A&T. A half-day dissemination workshop took place for about 80 actors from government, development agencies, private sector (companies, Euro Cham, Milk Association) and mass media.   September 2016   - The Ministry of Planning and Investment proposed a revision to Article 7.4 in the 2012 Advertisement Law to narrow the ban on advertisement of BMS for children from 24 to 12 months of age. The Ministry of Planning and Investment proposed using the fast-track method to approve these revisions, allowing less time for opposing viewpoints to be presented before a decision was made. - The strategic actors in Vietnam developed various strategies to respond to this threat on the Code. | 2012   - The Advertisement Law was approved. It banned advertising formula up to 24 months of age, complementary foods for children under 6 months of age as per the BMS code, feeding bottles and teats. - Regulations were strengthened and covered loopholes of the previous regulations (Decree 21), even beyond the International Code.   Dec 2014   - Decree 100/2014/ND-CP on marketing and use of feeding products for young children, feeding bottles, teats, and pacifiers was approved. The decree further specifies the Advertisement Law.   2015   - Trainings were carried out countrywide for the monitoring of the Code.   October 2016   - The government (economic committee) decided to not consider the proposal to revise the Advertisement Law. The law remained unchanged.   Nov 2016   - A circular to guide the implementation of Decree 100 is developed by the MCH department with support from partners. |
| **Burkina Faso** | Jan 2016  Small committee for the revision of a Decree for the Code  October 2016  Validation workshop for the Decree. A&T supported financially this activity. | October 2017  Draft of a Decree validated during a validation workshop |
| **Ethiopia** | 2016   - Support the Federal Ministry of Health (FMOH) and other partners in pushing for the implementation of the national BMS guideline. - Contribute to the bottleneck study being planned by Nutrition Development Partners (A&T commented on it). - Share A&T experience regarding the BMS in other countries through presentations and exchange visits to key stakeholders. - UNICEF supports a staff placement in The Ethiopian Food, Medicine and Health Care Administration and Control Authority (EFMHCA) - Bottleneck study TOR drafted | March 2016  The Infant Formula & Follow-up formula Directive was enacted by the EFMHCA (second directive that is part of the International Code)  The Code has been identified as a growing challenge and endorsed as an advocacy issue by donors who have capacity to influence the policy environment. |
